# Factors determining intention to quit tobacco: exploring patient responses visiting public health facilities in India

**DOI:** 10.1186/1617-9625-12-1

**Published:** 2014-01-20

**Authors:** Rajmohan Panda, Sudhir Venkatesan, Divya Persai, Mayur Trivedi, Manu Raj Mathur

**Affiliations:** 1 Public Health Foundation of India, 2nd Floor, PHD House, August kranti Marg, 4/2 Siri Institutional Area, New Delhi 110016, India; 2 Division of Epidemiology and Public Health, University of Nottingham, Room B110 Clinical Sciences Building, Nottingham City Hospital, Hucknall Road, Nottingham NG5 1 PB, UK; 3 Indian Institute of Public Health, Gandhinagar, Sardar Patel Institute Campus, Drive-in Road, Thaltej, Ahmadabad 380054, India

**Keywords:** Intention to quit, Motivating factors, Public health facilities, Tobacco, India

## Abstract

**Introduction:**

Intention to quit and setting a quit date are key steps in the process towards improving quit rates and are thus an integral part of tobacco cessation efforts. The present study examined various motivating factors of “intention to quit” and “setting a quit date” in patients visiting public health facilities in two states of India.

**Methods:**

A total of 1569 tobacco-users visiting public health facilities in 12 districts of the states of Andhra Pradesh and Gujarat were assessed through an interviewer-administered questionnaire. Bivariate and multivariable logistic regression was performed to assess the effect of socio-demographic characteristics, nicotine dependence, previous quit attempts and motivational factors on “intention to quit within 30 days” and “setting a quit date”.

**Results:**

Only 12% of patients intended to quit tobacco within 30 days and about 11% of them were ready to set a quit date. Respondents aged above 25 years were 53% less likely to quit tobacco within 30 days when compared to those below 25 years (95% Confidence Intervals [CI]: 0.22 to 0.99). Smokeless tobacco users were associated with an odds ratio (OR) of 2.05 (95% CI: 1.15 to 3.65) for “setting a quit date” when compared to smokers. Those with 1 to 5 previous quit attempts (in the past twelve months) were associated with an OR of 2.2 (95% CI: 1.38 to 3.51) for “intention to quit” and 2.46 (95% CI: 1.52 to 3.96) for “setting a quit date”. “Concern for personal health” and “setting an example for children” were associated with ORs of 3.42 (95% CI: 1.35 to 8.65) and 2.5 (95% CI: 1.03 to 6.03) respectively for “setting a quit date”.

**Conclusions:**

This study is amongst the first in India to explore factors associated with the “intention to quit” and “setting a quit date” among patients visiting public health facilities. Our findings suggest that socio-economic and individual-level factors are important factors depicting intention to quit and setting a quit date. We recommend the need for well-defined studies to understand the long term effects of factors influencing tobacco cessation for patients visiting public health facilities in India.

## Background

The tobacco epidemic is shifting rapidly from developed to developing countries. The recent report launched by the World Health Organization (2012) on mortality attributable to tobacco indicates towards a very high burden of deaths attributable to tobacco use in South East Asia region especially in India [1]. Following current tobacco use trends, 10 million deaths related to tobacco are predicted to occur worldwide by the year 2030, and about 7 in 10 of these deaths will be in low-income countries [2]. More than one third of India’s adult population (275 million persons) was estimated to be tobacco users in 2010. These persons comprised 68.9 million smokers, 163.7 million smokeless users and 42.3 million users of both products [3]. Cessation of tobacco use has the potential to provide the most immediate benefits of tobacco control and maximize the benefits in terms of preventable disease morbidity and mortality [4].

Intention to quit and setting a quit date is highly associated with attempting to quit tobacco and is one of the key steps in the process towards tobacco cessation [5]. However, as per Global Adult Tobacco Survey, (GATS), India (2009-10) survey, only 38% of smokers and 35% of smokeless tobacco users attempted to quit in the past 12 month period prior to the survey. Out of this, around 12% of smokers and 15% of smokeless tobacco users reported intention to quit tobacco in next one month following the GATS survey [3].

A number of factors affect the intention to quit and setting a quit date among tobacco users. Studies have shown that factors such as socio-economic status and education [6], physician’s advice to quit [7], tobacco risk perception [8] and nicotine dependence [9] are associated with the intention to quit and frequency of quit attempts.

Obtaining information on factors affecting the intention to quit and setting a quit date can contribute significantly to improving tobacco cessation activities at an individual as well as at the population level. Understanding patient interest in quitting, reasons for quitting, and associated factors may assist health care providers in addressing tobacco use in clinical settings, and aid the development and delivery of more effective nicotine-dependence treatment [10].

A study conducted in India revealed the need for detailed analysis on socio-demographic characteristics of people who attempted to quit tobacco [6]. To the best of our knowledge, there are no studies in Indian and South-Asian contexts which have examined specific factors affecting “setting of a quit date” among tobacco users and only a few studies have investigated the factors related to intention to quit among tobacco users in India [11,12]. The lack of such information is a significant constraint which limits the design of appropriate tobacco cessation programs in developing countries such as India. The purpose of our study was to examine various factors determining the “intention to quit” and “setting a quit date” among patients visiting public health facilities in two high tobacco burden states of India.

## Methods

### Study design & procedures

The study was a cross sectional survey conducted in 2012 among 1569 patients visiting public health facilities in 12 districts of Andhra Pradesh and Gujarat. Public health facilities are the cornerstones of health services- a first port of call to a qualified doctor of the public sector for the sick and those who directly report or referred for curative, preventive and promotive health care. The health facilities were selected through systematic random sampling. The study participants were selected through simple random sampling. Responses were collected by means of an interviewer administered questionnaire, which comprised five sections namely a) ‘Participant eligibility’, b) ‘Socio-demographic information’, c) ‘Tobacco use information’, d) ‘Tobacco counselling practices by healthcare providers,’ and e) ‘Motivation to quit and tobacco cessation’. Nicotine dependence for both, smoked and smokeless forms of tobacco was measured using the Fagerstrom Test for Nicotine Dependence (FTND). FTND is a valid self-reported measure of nicotine dependence [13].

### Recruitment of respondents and consent

The participant eligibility was determined on the basis of whether the respondent was adult (more than 18 years), sought services from health service providers, and consumed tobacco in some or the other form. Recruitment and data collection was done by trained researchers after the patients had exited from the consultation rooms and at locations away from the consultation rooms. Critically ill patients, those younger than 18 years, and those who did not give consent were excluded from the study.

### Dependent variables

The dependent variables that we used for this study were “Intention to quit within 30 days” and “setting a quit date”. Intention to quit was determined using the pre-validated Readiness to Quit Ladder [14]; a scale of items 1-10 (‘I have quit smoking’ to ‘I have decided not to quit smoking for my lifetime, and I have no interest in quitting’). Responses were captured as “yes” or “no”. A response of “yes” to “I definitely plan to quit in the next 30 days” and “I am ready to set a quit date” were used to indicate “Intention to quit within 30 days” and “setting a quit date” respectively. “No intention to quit” (also recorded from the 10-point quitting ladder) was used as the comparison category.

### Independent variables

#### Socio-demographic factors

The following socio-demographic measures were included in the analyses: age, sex, location (rural/urban), religion, community, marital status, level of education, occupation, and socio-economic status. Rural and urban stratification was done on the basis of census of India data [15]. Age was divided into two categories: less than 25 years and more than 25 years, a priori decision was made to divide age into two categories, and not include it as a continuous variable to avoid any potential collinearity with ‘age at initiation of the habit’. Respondents’ highest level of education was measured according to four categories i.e. uneducated, less than primary, primary but less than secondary and secondary and above. Socio-economic status was captured by ‘below and above poverty line’ status. Religion was recorded as either ‘Hindu, Muslim, Christian, Sikh, Jain or others’. Caste of an individual is the basis of social hierarchical organisation in India. The government of India has identified the castes occupying the lowest rung of social hierarchy as the most socially disadvantaged and indigenous people, and classified them as scheduled castes (SCs) and the scheduled tribes (STs) respectively. In addition some castes were identified as socially disadvantaged and classified as “Other Backward Class” (OBCs) [16]. Other Backward Class (OBC) is a collective term used by the Government of India to classify castes which are educationally and socially disadvantaged [17].

#### Tobacco usage and addiction

Tobacco usage, age at initiation of the habit, FTND scores for smoked and smokeless forms of tobacco, use of other addictive substances (alcohol, opium etc.) and the number of previous quit attempts in past 12 months were taken as independent variables. Each FTND component was recorded separately, and subsequently a compsite FTND was obtained for smoked and smokeless forms of tobacco.

#### Previous quitting experience

Previous quitting experience of smokers consisted of ‘time since most recent serious quit attempt’ and ‘motivational factors for previous quit attempt’. The latter comprises a series of nine binary variables which includes concern for personal health, concern about effect on non-smokers, price of cigarettes/bidis, smoke-free laws, advertisements on health effects of smoking, health warning labels on cigarette packages, setting an example for children and family disapproval of smoking.

### Data analysis

Statistical analyses were carried out using Stata 12.0 (StataCorp, 2011. Stata Statistical Software: Release 12.) [18]. Chi-square tests were conducted to examine bivariate differences between socio-demographic characteristics, prevalence of tobacco, nicotine dependence and “ intention to quit” and “setting a quit date”. “Individual factors” and “Tobacco usage and addiction” were the two groups of independent variables that were included in the bivariate analyses. For continuous variables, independent samples t-test was used to test the association. Those variables that were statistically significant (p-value <0.05) in the bivariate analyses were included in a multivariable logistic regression model to examine factors that might affect the “intention to quit” and “setting a quit date”. Several demographic factors were included in the analyses including sex, age group, religion, urban/rural area, socio-economic status and education level. The data for this study were collected from 12 different districts in India and to take into account this clustered nature of the data, the multivariable model was devised as a hierarchical model with ‘district’ introduced as a random intercept. A sub-group analysis was conducted among respondents who were smokers and who had made at least one quit-attempt, in order to investigate the role of “previous quitting experience” on the outcomes of “intention to quit” and “setting a quit date”.

### Ethical considerations

A written consent was taken from all the respondents before conducting the interviews. Prior permission from the state and district authorities was taken before conducting the survey in their premises. The ethical clearance was obtained from institutional ethical committee (IEC no 65/60).

## Results

### Demographic characteristics of the sample

Approximately equal numbers of patients were sampled in each state, with a higher proportion of rural residents (75%) compared to urban residents (25%). A response rate of 90% was observed. A total 1,569 patients were sampled with the proportion of males (80%) being much higher than females (20%). Majority of the respondents had some level of education (56%) with the proportion of the population under 25 years within each level of education increasing steadily (3%, 10%, 18% and 30% of the group being under 25 years for each of the four levels of education respectively). More than three quarters of the respondents were married (82%) and employed (84%). About 47% of respondents were smokeless tobacco users, 45% of them were smoked tobacco users and 4% used dual forms of tobacco. Table 1 illustrates the differences in baseline characteristics among respondents with “intention to quit” and “setting a quit date”.

**Table 1:** Characteristics of patients visiting public health facilities (n = 1569)

	**Intention to quit within 30 days (Yes = 183; No = 820)**		**Setting a quit date (Yes = 172; No = 820)**	
**Characteristics**	**N (%)**	**p-value**	**N (%)**	**p-value**
**Demographic and health condition**				
**Age (years)**				
≤ 25 years	**42 (23)**	**<0.001**	**30 (17)**	**<0.001**
>25 years	**141 (77)**		**145 (83)**	
***Sex***				
Male	146 (80)	0.621	125 (71)	0.057
Female	37 (20)		50 (29)	
***Location***				
Rural	133 (81)	0.211	**108 (69)**	**0.038**
Urban	31 (19)		**49 (31)**	
***Religion***				
Hindu	159 (87)	0.558	146 (84)	0.429
Muslim	16 (9)		16 (9)	
Christian	7 (4)		11 (6)	
Others (Sikh, Jain & Buddhism)	1 (0.6)		1(1)	
***Caste***				
SC	31 (17)	0.073	40 (24)	0.385
ST	26 (14)		25 (15)	
OBC	73 (40)		68 (40)	
General	51 (28)		37 (22)	
***Marital status***				
Single	41 (22)	0.062	35 (20)	0.277
Married	142 (78)		140 (80)	
***Education***				
Uneducated	**51 (29)**	**<0.001**	**55 (33)**	**<0.001**
Less than primary	**58 (32)**		**40 (24)**	
Primary but less than secondary	**49 (27)**		**48 (29)**	
Secondary and above	**22 (12)**		**24 (14)**	
***Occupation***				
Employed	175 (96)	0.001	2 (1)	0.58
Unemployed	**7 (4)**		173 (99)	
***Socio-economic status***				
Below poverty line	**82 (45)**	**0.007**	99 (59)	0.097
Above poverty line	**99 (55)**		70 (41)	
***Self-reported health***				
Poor	**31 (17)**		**28 (16)**	**<0.001**
Fair	**82 (45)**	**<0.001**	**57 (33)**	
Good	**70 (38)**		**90 (51)**	
**Tobacco Usage & Addiction**				
***Forms of tobacco***				
Smoked tobacco	**66 (36)**	**<0.001**	**45 (26)**	**<0.001**
Smokeless tobacco	**110 (60)**		**116 (66)**	
Both	**7 (4)**		**14 (8)**	
***Age at initiation (years)***	21.98 ± 9.58	0.82	22.16 ± 9.89	0.65
***FTND score for smoked tobacco (0 - 8)***	4.19 ± 1.25	0.062	**4.14 ± 1.21**	**0.025**
***FTND score for smokeless tobacco (0 - 7)***	**3.53 ± 0.11**	**<0.001**	**3.47** ± **1.45**	**<0.001**
***Other substances (Alcohol, opium etc*.)**				
Yes	**22 (12)**	**<0.001**	**19 (11)**	**<0.001**
No	**160 (88)**		**156 (89)**	
***Previous quit attempts***				
No. of quit attempts in past 12 months				
0	**77 (47)**		**74 (43)**	
1 to 5	**83 (51)**	**<0.001**	**81 (47)**	**<0.001**
5 to 10	**2 (1)**		**14 (8)**	
>10	**2 (1)**		**2 (1)**	

Bold text indicates statistically significant at p < 0.05.

### Intention to quit tobacco

Overall, only 12% (183) of the total study population intended to quit within 30 days, while 52% (820) reported no intention to quit tobacco and analyses were performed on this group of 1,003 patients. Of the demographic factors that were included in multivariable logistic regression analyses, only four of the factors showed significant associations on the “intention to quit”: tobacco products, age, employment, and previous quit attempts. Table 2 presents the odds ratios of “intention to quit” and “setting a quit date” for each of the socio-demographic and other factors. Findings indicate that smokeless tobacco users had higher intention to quit as compared to smokers (adjusted OR = 1.7, 95% CI = 1.04-2.73). Respondents aged above 25 years were 53% less likely to intend to quit tobacco within 30 days (OR: 0.47; 95% CI: 0.22 to 0.99) when compared to those aged 25 years and younger. Interestingly, those in employment were associated with an 85% reduction in odds, when compared to the unemployed for “intention to quit”. In addition, number of previous quit attempts (in the past twelve months) was an important factor of intention to quit within 30 days, with ‘1 to 5 quit attempts’ showing the strongest association; estimates for the other categories did not reveal statistically significant associations. Analysis revealed no significant association between “Intention to quit” and motivational factors such as concern for personal health, setting an example for children, price of cigarettes/bidis and warning labels on cigarette packages.

**Table 2:** Multivariable logistic regression results of factors affecting intention to quit and setting a quit date among patients visiting public health facilities

	**Intention to quit within 30 days (n = 1003)**		**Ready to set quit date (n = 995)**	
**Characteristics**	**Unadjusted OR (95% CI)**	**Adjusted OR (95% CI)**	**Unadjusted OR (95% CI)**	**Adjusted OR (95% CI)**
***Demographic***				
***Age (years)***				
≤25 years	Reference	Reference	Reference	Reference
**>25 years**	**0.31 (0.16 to 0.59)**	**0.47 (0.22 to 0.99)**	**0.46 (0.26 to 0.83)**	0.70 (0.34 to 1.43)
***Education***				
Uneducated	Reference	Reference	Reference	Reference
Less than primary	**1.74 (1.01 to 3.00)**	1.30 (0.71 to 2.36)	1.08 (0.63 to 1.86)	0.99 (0.53 to 1.85)
Primary but less than secondary	1.79 (0.99 to 3. 21)	1.27 (0.71 to 2.36)	1.63 (0.95 to 2.77)	1.09 (0.57 to 2.07)
Secondary and above	**2.32 (1.04 to 5.15)**	1.39 (0.56 to 3.43)	**2.27 (1.17 to 4.40)**	1.59 (0.72 to 3.50)
***Work***				
Unemployed	Reference	Reference	NS	NS
Employed	**0.08 (0.01 to 0.38)**	**0.15 (0.03 to 0.76)**	NS	NS
***Location***				
Rural	NS	NS	Reference	Reference
Urban	NS	NS	1.53 (0.95 to 2.46)	1.17 (0.69 to 1.99)
***Socio-economic status***				
Above PL	Reference	Reference	NS	NS
Below PL	**0.51 (0.32 to 0.83)**	0.63 (0.37 to 1.06)	NS	NS
***Self-reported health***				
Poor	Reference	Reference	Reference	Reference
Fair	1.08 (0.61 to 1.92)	1.20 (0.65 to 2.21)	0.73 (0.40 to 1.34)	0.61 (0.31 to 1.22)
Good	1.32 (0.69 to 2.53)	1.17 (0.56 to 2.45)	1.81 (0.99 to 3.29)	1.23 (0.61 to 2.49)
***Form of tobacco***				
Smoked tobacco	Reference	Reference	Reference	Reference
Smokeless tobacco	**1.69 (1.04 to 2.73)**	1.22 (0.71 to 2.09)	**2.52 (1.58 to 4.03)**	**2.05 (1.15 to 3.65)**
Both	0.52 (0.19 to 1.41)	0.43 (0.15 to 1.28)	**2.16 (1.03 to 4.52)**	1.31 (0.54 to 3.22)
***Other substances besides tobacco***				
No	Reference	Reference	Reference	Reference
Yes	1.01 (0.56 to 1.81)	0.95 (0.50 to 1.81)	0.91 (0.51 to 1.64)	0.85 (0.42 to 1.69)
***FTND score for smoked tobacco***	NS	NS	0.98 (0.85 to 1.14)	1.10 (0.91 to 1.34)
***FTND score for smokeless tobacco***	1.00 (0.84 to 1.19)	0.99 (0.82 to 1.20)	0.95 (0.81 to 1.11)	0.95 (0.80 to 1.13)
***No. of quit attempts in past 12 months***				
0	Reference	Reference	Reference	Reference
1 to 5	**2.28 (1.45 to 3.56)**	**2.20 (1.38 to 3.51)**	**2.73 (1.77 to 4.23)**	**2.46 (1.52 to 3.96)**
5 to 10	0.44 (0.08 to 2.51)	0.43 (0.06 to 3.02)	**7.92 (3.25 to 19.28)**	**7.50 (2.82 to 19.96)**
>10	0.70 (0.14 to 3.43)	0.87 (0.17 to 4.45)	1.00 (0.20 to 4.90)	1.20 (0.23 to 6.39)

NS: Not significant in the bivariate analyses.

Bold text indicates statistically significant at p < 0.05.

#### Setting a quit date

About 1% (175) of the total study population were ready to set a quit date while 52% (820) reported no intention to quit tobacco. Subsequent analyses were performed in this group of 995 patients. Contrary to the “intention to quit” category, an overwhelming majority of respondents (99%) in this category were unemployed; however this association was not statistically significant (*p-value = 0.58*). Results of the multivariable analysis suggest that smokeless tobacco users were about two times more likely to set a quit date when compared to smokers (OR:2.05; 95% CI: 1.15 to 3.65). Table 3 illustrates that previous quit attempts (in the past twelve months) was a strong factor of setting a quit date- ‘1 to 5 quit attempts’ was associated with an OR of 2.46 (95% CI: 1.52 to 3.96) along-with ‘5 to 10 quit attempts’ was associated with an OR of 7.50 (95% CI: 2.82 to 19.96). Findings suggest that those who attempted to quit in less than six months were about three times more likely to “set a quit date” than those respondents who attempted to quit in more than six months of duration. Such association were not observed in “intention to quit”. Data suggests that “concern for personal health” (OR 3.42, 95% CI: 1.35-8.65) and “setting an example for children” (OR 2.50, 95% CI: 1.03-6.03) were the two motivational factors for “setting a quit date”. These factors were not found significant for the “intention to quit” outcome.

**Table 3:** Factors of intention to quit smoking and setting a quit date

	**Intend to quit smoking within 30 days (n = 260)**		**Ready to set quit date**	
			**(n = 274)**	
**Characteristics**	**OR (95% CI)**	**p-value**	**OR (95% CI)**	**p-value**
***Previous quitting experience***				
***Time since most recent serious quit attempt***				
>6 months	Reference		Reference	
1 – 6 months	**4.40 (1.26 to 15.42)**	**0.021**	**2.96 (1.22 to 7.17)**	**0.016**
***Motivational factors***				
Concern for personal health				
No/A little	Reference		Reference	
Yes, strongly	1.60 (0.59 to 4.33)	0.357	**3.42 (1.35 to 8.65)**	**0.01**
Concern about effect on non-smokers				
No/A little	Reference		Reference	
Yes, strongly	0.78 (0.19 to 3.13)	0.723	0.82 (0.22 to 3.01)	0.763
Society’s disapproval of smoking				
No/A little	Reference		Reference	
Yes, strongly	3.50 (0.82 to 15.00)	0.092	2.69 (0.64 to 11.23)	0.175
Price of cigarettes/bidis				
No/A little	Reference		Reference	
Yes, strongly	0.93 (0.21 to 4.16)	0.926	2.79 (0.92 to 8.49)	0.071
Smoke free laws				
No/A little	Reference		Reference	
Yes, strongly	1.07 (0.22 to 4.18)	0.932	1.59 (0.42 to 6.02)	0.491
Advertisements/information on health risks of smoking				
No/A little	Reference		Reference	
Yes, strongly	1.94 (0.49 to 7.72)	0.345	2.83 (0.76 to 10.61)	0.123
Health warnings labels on cigarette packages				
No/A little	Reference		Reference	
Yes, strongly	1.41 (0.42 to 4.75)	0.582	0.37 (0.09 to 1.56)	0.176
Setting an example for children				
No/A little	Reference		Reference	
Yes, strongly	0.84 (0.29 to 2.46)	0.75	**2.50 (1.03 to 6.03)**	**0.042**
Family disapproval of smoking				
No/A little	Reference		Reference	
Yes, strongly	1.37 (0.47 to 4.00)	0.57	0.64 (0.21 to 1.94)	0.426

ORs adjusted for all variables mentioned in Table 3.

Bold text indicates statistically significant at p < 0.05.

### Nicotine dependence and intention to quit & setting a quit date

Findings suggest that nicotine dependence for smoking and smokeless tobacco was low to moderate with FTND score of 4.2 and 3.5 respectively. No significant association was noticed between FTND score and “intention to quit” as well as “setting a quit date” among the respondents.

## Discussion

The present study aimed to examine factors affecting the “intention to quit” and “setting a quit date” was limited to patients visiting health facilities in two states in India. Therefore, these results are not representative of tobacco users in the country as a whole.

Our findings in patients visiting health facilities revealed that “intention to quit” was comparable to the findings of other Indian studies [19] and that observed in GATS India survey conducted in 2010 in which about 12% tobacco users intended to quit in next one month of the survey. GATS India is a nationally representative survey of key indicators in tobacco control in India [3]. Our findings indicates that “intention to quit” was low when compared to the International Tobacco Control study findings in other Asian countries such as Malaysia and Thailand in which 58% and 30% of tobacco users intended to quit respectively [20]. “Intention to quit” in our study was also low when compared to that reported in China (24% of smokers across six states in 2009 planned to quit in next 6 months) and other developed countries with 65-81% having any intention to quit at some point in the future [21]. This finding underscores the need for greater effort to be made to stimulate interest in quitting among tobacco users in India.

In contrast to the findings of an Indian study [12], our findings indicate that users of smokeless tobacco were more likely to depict an intention to quit and setting a quit date as compared to smokers. This fact is promising in consideration of the high burden of smokeless tobacco in India.

Our study revealed that patients who were educated more than secondary level had higher intention to quit and setting a quit date as compared to those who were educated less than secondary level. This finding is consistent to the findings of an Indian study conducted by Srivastava et.al. [6]. The GATS 2009-10, India data also revealed a similar correlation between educational level and intention to quit. We find that higher number of employed respondents “intend to quit” tobacco as compared to unemployed respondents which is in accord with previous study conducted in other developing countries such as Thailand and Malaysia [20].

A study conducted in India suggests that male tobacco users had high odds of quit attempts compared to female [7]. However, no sex difference was observed in “intention to quit” and “setting a quit date” in our study. With regard to habitat it has been reported that tobacco users residing in urban areas had greater intention to quit as compared to rural residents [13]. In our study rural residents were more likely to “set a quit date” as compared to urban counterparts. In the present study, we found that “intention to quit” was higher among patients who have high income as compared to those with low income. There is in accordance to previous studies conducted in Indian and South-Asian region which suggests that tobacco users with lower socio-economic status were associated with no intention to quit [7,21].

Quitting tobacco is a dynamic process involving repeated attempts to quit [22]. An important finding in this study was the strong association of previous quit attempts with the “intention to quit” and “setting a quit date”. Similar findings were observed in other studies across the globe [23,24]. This is very much in line with the fact that tobacco users require multiple attempts to quit before quitting [25]. Health care providers should give due consideration to past quitting experience of patient while providing tobacco cessation intervention.

The respondents in the study reported low to moderate nicotine dependence. Studies have shown that the more dependent a person is on nicotine, the more difficulty they have in quitting [26,27]. Findings of our study reported that patients with low to moderate dependence were likely to have an “intention to quit” and were ready to “set a quit date”.

Majority of the patients in our study rated their health to be fair and good. Similar observations were made in an Indian study in which majority of smokers perceived that they are in good health [19]. In the present study we observed that self rated health status of patients was not associated with the “intention to quit” and “setting a quit date”. This finding is in contrast to the findings of the study done by Yang et.al. (2010) in China which suggests that intention to quit was higher among smokers who felt that smoking had damaged their own health [28]. In our study, majority of patients perceived that they are in good health and thus health status was not a motivating factor for quitting tobacco.

Finally, we also assessed the effect of different motivational factors on the “intention to quit” and “setting a quit date” among smokers. Findings of our study suggests that concern of personal health and setting an example for children is as an important motivator for “setting a quit date”. This finding is consistent to the other studies which states that most important reason for quitting smoking was general health concern and setting an example for children [26,29]. Our findings reveal that smoke-free laws and health warnings on cigarette packages appeared to be a weaker motivator for quitting among patients in both states. Similar observations were made in a study conducted in India which states that pictorial health warnings on cigarette packages are ineffective in motivating smokers to quit [30]. Several tobacco control measures were introduced in India during last couple of years including a ban on tobacco products advertising, ban on smoking at public places, text and pictorial health warnings on tobacco products [31]. There is a need to evaluate the implementation status and effectiveness of these tobacco control policies and programs to understand their effect on tobacco cessation.

Our study suggests that socio-demographic factors such as education, age, location, tobacco products, and number of quit attempts are important factors determining “intention to quit” and “setting a quit date”. When we examined motivational factors affecting “setting a quit date” which demonstrates action stage in tobacco cessation we found that only concern for personal health, and setting an example for children determine the outcome of “setting a quit date”. Other factors such as smoke-free laws, advisements on health risks of smoking and warnings on cigarette packages were not reported as significant factors determining “setting a quit date.”

### Limitations

The present sample was limited to patients visiting health facilities in two states in India. Therefore, these results are not representative of tobacco users in the country as a whole. Another limitation of the current study was the small sample size of respondents who had intention to quit which meant that a more elaborate model exploring the incremental effect of important covariates such as age, along with age at initiation, could not be possible. In addition, because of cross-sectional design of the study, we were only able to explore associations between our measures without addressing possible causal relationships between “intention to quit” and “setting a quit date” and our explanatory variables. The present study relied on self-reported responses and may be subject to recall bias. Nevertheless, this study can provide direction to future research on factors that would influence tobacco cessation interventions in health care settings and help set a baseline for understanding patient behaviour in tobacco cessation in primary health care.

## Conclusions

This study is important as it is one among its kind in India which examined patients’ responses to factors influencing “intention to quit” and “setting a quit date”. Our findings suggest that socio-economic factors such as age & education and individual-level factors such as nicotine dependence, previous quit attempts and concern for personal health were associated with intention to quit and setting a quit date. Our study highlights the need of targeted cessation interventions amongst the vulnerable population to motivate them to quit tobacco use. Identifying the determinants of quit intentions will provide possibilities for shaping effective programs for increasing quitting among tobacco users in India. There is a paucity of data for understanding factors influencing cessation practises in patients as well as the general population; we recommend the need for well-designed studies to examine the long term effects of the factors of tobacco cessation in both India as well as in other low and middle income settings. This will help in designing appropriate programs for tobacco cessation in such settings.

## Abbreviations

FTND: Fagerstrom Test for Nicotine Dependence; OBC: Other Backward Class; SC: Schedule caste; ST: Schedule tribe.

## Competing interests

The authors declared that they have no competing interests.

## Authors’ contributions

RP, SV and DP conceptualized and planned the overview of the paper. RP was involved in designing and conducting the study as well as the design of the paper. RP wrote the introduction and shaped the discussion piece. SV led the data analysis and wrote the results. DP contributed to the interpretation and writing of the results and discussion. MM and MT reviewed the manuscript and provided inputs relating to technical quality of the paper. All authors read and approved the final manuscript.

## Authors’ information

Sudhir Venkatesan, Divya Persai, Mayur Trivedi and Manu Raj Mathur are co-authors.
